# Cardiovascular Biomarkers: Tools for Precision Diagnosis and Prognosis

**DOI:** 10.3390/ijms26073218

**Published:** 2025-03-30

**Authors:** Vasudeva Reddy Netala, Tianyu Hou, Yanbo Wang, Zhijun Zhang, Sireesh Kumar Teertam

**Affiliations:** 1 School of Chemical Engineering and Technology, North University of China, Taiyuan 030051, China; vasunuc1922@gmail.com (V.R.N.); tyhou@nuc.edu.cn (T.H.); testinlife@foxmail.com (Y.W.); 2 Department of Dermatology, School of Medicine and Public Health, University of Wisconsin-Madison, Madison, WI 53705, USA

**Keywords:** cardiovascular biomarkers, cardiac troponins, miRNAs, heart failure biomarkers, TNFα, IL-6, copeptin and endothelin-1

## Abstract

The present study provides a detailed review of cardiovascular biomarkers critical for the diagnosis, prognosis, and pathophysiology of cardiovascular diseases, the leading cause of global morbidity and mortality. These biomarkers aid in detecting disease onset, progression, and therapeutic responses, providing insights into molecular mechanisms. Enzyme markers like AST, CK-MB, LDH, CA-III, and HBDH are pivotal for detecting myocardial injury during acute events. Protein markers such as CRP, H-FABP, and MPO shed light on inflammation and oxidative stress. Cardiac Troponins, the gold standard for myocardial infarction diagnosis, exhibit high specificity and sensitivity, while IMA and GPBB indicate ischemia and early myocardial damage. Peptide markers, including BNP and NT-proBNP, are crucial for heart failure diagnosis and management, reflecting ventricular stress and remodeling. Novel peptides like MR-proANP and MR-proADM aid in assessing disease severity. Lipid markers such as lipoprotein-associated phospholipase A2 and oxylipins provide insights into lipid metabolism and atherosclerosis. Inflammatory and stress-related biomarkers, including TNFα, IL-6, GDF-15, and Pentraxin 3, illuminate chronic inflammation in CVDs. Hormonal markers like copeptin and endothelin-1 highlight neurohormonal activation, while emerging markers such as ST2, galectin-3, PAPP-A, and TMAO elucidate fibrosis, remodeling, and metabolic dysregulation. The inclusion of microRNAs and long non-coding RNAs represents a breakthrough in biomarker research, offering sensitive tools for early detection, risk stratification, and therapeutic targeting. This review emphasizes the diagnostic and prognostic utility of these biomarkers, advancing cardiovascular care through personalized medicine.

## 1. Introduction

Cardiovascular diseases (CVDs) remain the leading cause of morbidity and mortality worldwide, accounting for approximately 17.9 million deaths annually [1]. The growing burden of CVDs is driven by lifestyle changes, increasing prevalence of risk factors, such as hypertension, diabetes, obesity, and smoking, as well as genetic predisposition. CVDs not only impact individual health but also impose substantial economic burdens on healthcare systems globally [2]. Early diagnosis and timely intervention play a crucial role in improving patient outcomes and reducing mortality rates associated with cardiovascular conditions. Biomarkers have emerged as essential tools in the early detection, risk stratification, and management of various CVDs, enabling precise and timely therapeutic strategies. Advances in biomarker research have led to the identification of novel molecular indicators that enhance diagnostic accuracy and predictive capabilities. These biomarkers help in distinguishing between different cardiac conditions, monitoring disease progression, and guiding therapeutic interventions. Furthermore, the development of high-sensitivity assays has improved the detection limits of traditional biomarkers, allowing for earlier recognition of myocardial injury. The integration of biomarker analysis with emerging technologies such as artificial intelligence and machine learning holds promise for further refining risk assessment and clinical decision-making [3,4,5]. Continued research and validation of new biomarkers are essential to expanding their clinical utility and improving cardiovascular health outcomes globally.

## 2. Cardiovascular Diseases

**Figure 1:**
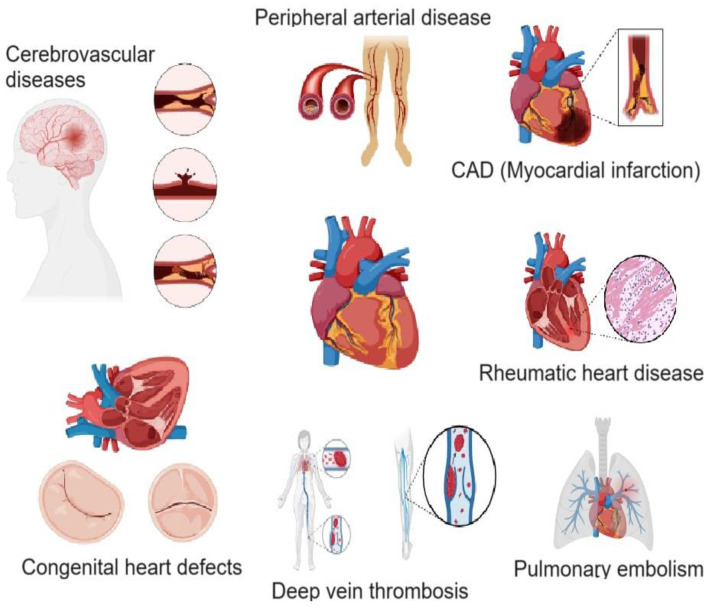
Schematic illustration of various cardiovascular diseases. Figure was created using app.biorender.com.

CVDs encompass a broad spectrum of disorders affecting the heart and blood vessels. Figure 1 represents different types of CVDs including coronary artery disease (CAD), peripheral artery disease (PAD), deep vein thrombosis (DVT) and pulmonary embolism (PE), rheumatic heart disease (RHD), congenital heart defects (CHD), and cerebrovascular diseases. Among these, CAD is the most prevalent, characterized by the narrowing or blockage of coronary arteries due to atherosclerosis, which can lead to myocardial infarction (MI). The progression of CAD can cause angina, arrhythmias, and heart failure if left untreated. Lifestyle modifications, medications, and interventional procedures such as angioplasty or coronary artery bypass grafting (CABG) are crucial in its management [6]. PAD involves the obstruction of blood flow in the peripheral arteries, increasing the risk of ischemic limb conditions. Symptoms often include claudication, pain, and numbness in the affected limbs. Severe cases may progress to critical limb ischemia, necessitating surgical interventions like revascularization or even amputation [7]. DVT and PE result from the formation of blood clots in deep veins, potentially leading to life-threatening complications if the clot dislodges and travels to the lungs. DVT typically presents with leg swelling, pain, and redness, while PE may cause chest pain, shortness of breath, and hemodynamic instability. Prompt anticoagulation therapy and thrombolytic interventions are essential to prevent fatal outcomes [8]. RHD arises from untreated streptococcal infections, leading to chronic heart valve damage. It primarily affects the mitral and aortic valves, causing stenosis or regurgitation, which can progress to heart failure over time. Prevention through early antibiotic treatment of streptococcal infections is key, while severe cases may require valve repair or replacement surgery [9]. CHDs refer to structural abnormalities present at birth, affecting normal heart function. These defects range from simple atrial or ventricular septal defects to complex conditions such as Tetralogy of Fallot or transposition of the great arteries. Advances in pediatric cardiology and surgical techniques have significantly improved the prognosis and quality of life for affected individuals [10]. Additionally, cerebrovascular diseases, including stroke and transient ischemic attacks (TIAs), result from impaired blood flow to the brain, leading to severe neurological consequences. Ischemic stroke is the most common type, often caused by thromboembolism, while hemorrhagic stroke results from ruptured blood vessels. Early intervention with thrombolysis, thrombectomy, or neuroprotective strategies is crucial in minimizing brain damage and improving recovery outcomes [11].

## 3. Diagnosis of CVDs and the Role of Biomarkers

The accurate and timely diagnosis of CVDs is crucial for effective management and improved patient outcomes. Various diagnostic modalities, including electrocardiography (ECG), echocardiography, angiography, and cardiac MRI, provide valuable structural and functional insights of CVDs [12]. However, biomarkers have revolutionized CVD diagnosis by offering a minimally invasive, highly sensitive, and rapid means of detecting myocardial injury, inflammation, and hemodynamic stress. Cardiac biomarkers such as troponins (cTnI and cTnT), creatine kinase MB (CK-MB), brain natriuretic peptide (BNP), and high-sensitivity C-reactive protein (hs-CRP) play a pivotal role in diagnosing acute coronary syndromes (ACS), heart failure, and other cardiovascular conditions [13]. Emerging biomarkers, including microRNAs, myeloperoxidase (MPO), and growth differentiation factor-15 (GDF-15), hold promise for enhancing early detection and risk assessment [14]. The integration of biomarkers with advanced imaging techniques and machine learning approaches further strengthens the accuracy of CVD diagnostics, paving the way for personalized medicine in cardiology. The present study significantly provides a comprehensive review of various cardiac biomarkers, discussing their clinical utility, diagnostic accuracy, and potential applications. Additionally, it highlights the evolving landscape of biomarker research and the future directions for improving cardiovascular diagnostics.

## 4. Cardiac Biomarkers

Cardiovascular disease (CVD) biomarkers are crucial biological indicators that help in the early detection, diagnosis, and management of heart-related conditions. These biomarkers, which include proteins, enzymes, hormones, and genetic markers, provide valuable insights into the physiological and pathological processes underlying CVDs. By identifying specific biomarkers associated with cardiovascular events, healthcare providers can more accurately assess disease risk, monitor disease progression, and tailor personalized treatment strategies to improve patient outcomes.

### 4.1. Aspartate Aminotransferase (AST)

Originally identified in various tissues, including the liver, heart, skeletal muscle, brain, and kidney, AST has been utilized as a diagnostic marker since 1954. Its presence in cardiac tissue makes it particularly useful in assessing myocardial injury, although its diagnostic specificity for heart conditions may be limited due to its expression in other tissues. Elevations in AST levels often accompany MI, liver diseases, and muscular disorders, necessitating its interpretation alongside other cardiac biomarkers. The AST-to-ALT ratio is also considered in differentiating cardiac and hepatic pathologies, improving diagnostic accuracy [15].

### 4.2. Creatine Kinase MB (CK-MB)

Creatine kinase exists in three isoforms: CK-MB, primarily found in heart muscle but also in small amounts in skeletal muscle; CK-MM, predominantly in skeletal muscle; and CK-BB, located in the brain. Elevated CK-MB levels are indicative of MI, helping to differentiate between cardiac and skeletal muscle damage. Its incorporation into diagnostic panels in 1979 marked a significant advancement in MI diagnosis. The rise and fall of CK-MB levels provide insight into the timing of myocardial injury, peaking within 24 h and returning to baseline within 48–72 h. Although widely used, CK-MB has been partially replaced by troponins due to their superior sensitivity and specificity for cardiac injury [16].

### 4.3. Lactate Dehydrogenase (LDH)

LDH, with its five isozymes expressed in distinct organs, has been utilized since the 1960s to diagnose acute myocardial infarction, particularly as a marker of heart dysfunction. LDH1, expressed in cardiomyocytes, underscores its relevance in assessing cardiac injury. Despite its widespread use, LDH’s diagnostic specificity may be limited by its expression in various tissues. LDH isoenzyme analysis, particularly the LDH1/LDH2 ratio, enhances its diagnostic value in myocardial infarction detection. However, due to its delayed elevation and lower specificity, LDH has been largely replaced by cardiac troponins in modern clinical practice [17].

### 4.4. Carbonic Anhydrase III (CA-III)

CA-III is a cytosolic enzyme abundantly found in cardiac muscles, where it plays crucial roles in maintaining cellular homeostasis and protecting against oxidative stress. Its antioxidative properties are well documented, primarily attributed to its ability to scavenge oxygen radicals and mitigate oxidative injury [18]. CA-III serves as a cardiac biomarker due to its specific localization and functions within cardiac tissues. It participates in the regulation of intracellular pH, which is critical for the proper functioning of cardiac muscle cells. This enzyme helps in maintaining the acid–base balance, which is essential for the contraction and relaxation cycles of the heart. In addition to its role in oxidative stress mitigation and pH regulation, CA-III has been implicated in various physiological processes within the heart. It contributes to the overall antioxidant defense system of cardiac cells, protecting them from damage caused by reactive oxygen species (ROS) generated during normal cellular metabolism or under stress conditions [19]. Research has shown that CA-III levels can be altered under pathological conditions such as cardiovascular diseases, making it a potential diagnostic and prognostic marker. Its expression and activity can be influenced by factors such as ischemia, hypoxia, and oxidative stress, reflecting the dynamic response of the heart to various physiological and pathological stimuli [20].

### 4.5. Heart-Type Fatty Acid-Binding Protein (H-FABP)

**Figure 2:**
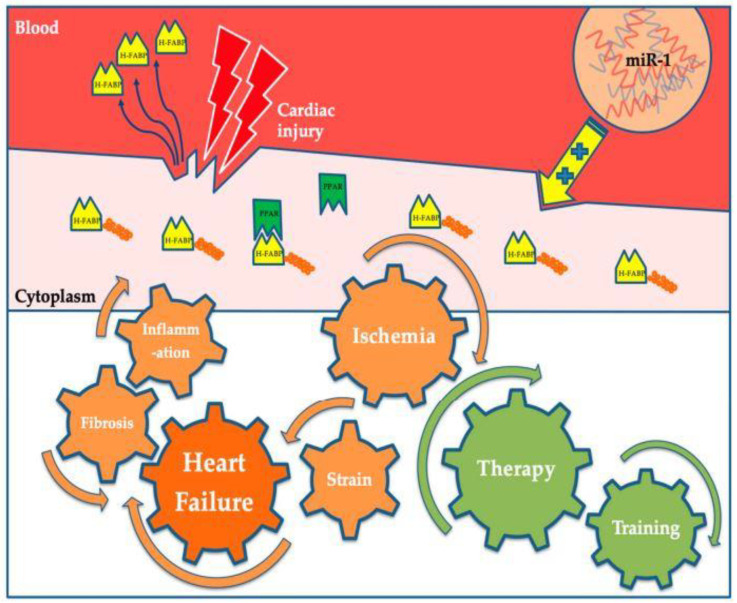
Schematic representation of the role of H-FABP in cardiac injury, metabolism, and therapeutic modulation. Under physiological conditions, H-FABP functions as a transport protein in cellular metabolism, reversibly binding fatty acids and activating peroxisome proliferator-activated receptors (PPARs), thereby contributing to lipid metabolism and energy homeostasis. The expression of H-FABP is regulated by microRNA-1 (miR-1). In response to cardiac injury, H-FABP is rapidly released into the bloodstream, where it can be quantified as a biomarker. Increased H-FABP levels are associated with ischemia, inflammation, fibrosis, and strain, ultimately leading to heart failure. Physical training and pharmacological interventions, such as anti-tachycardic therapy, have been shown to reduce plasma H-FABP levels, supporting their role in therapy. Figure is adapted from Rezar et al. [23].

H-FABP is a small cytoplasmic protein (12–15 kDa) abundant in tissues with active fatty acid metabolism, particularly in the heart. H-FABP plays a crucial role in myocardial homeostasis, facilitating the intracellular transport of insoluble fatty acids essential for the heart’s energy needs. Approximately 50–80% of the heart’s energy is derived from lipid oxidation, underscoring the importance of H-FABP in cardiac metabolism [21]. Following myocardial cell damage, H-FABP diffuses rapidly through the interstitial space, appearing in the circulation as early as 90 min after the onset of symptoms. It reaches its peak concentration within six hours. This rapid appearance in the bloodstream makes H-FABP an excellent candidate marker for early detection of myocardial injury [22]. H-FABP was first identified as a potential biomarker for myocardial injury in 1988, and since then, there has been growing interest in its diagnostic and prognostic utility. Studies have demonstrated that H-FABP is exceptionally sensitive in the first few hours after myocardial injury and during reperfusion injury, highlighting its potential to detect acute myocardial damage soon after its onset. H-FABP’s rapid appearance in the circulation following myocardial injury, coupled with its high sensitivity, makes it a valuable biomarker for the early detection of myocardial injury. Its role in fatty acid metabolism within cardiomyocytes further underscores its importance in myocardial homeostasis and as a diagnostic tool in clinical settings [23]. Figure 2 illustrates the physiological and pathophysiological roles of H-FABP, highlighting its significance in both normal cardiac function and myocardial injury.

### 4.6. C-Reactive Protein (CRP)

**Figure 3:**
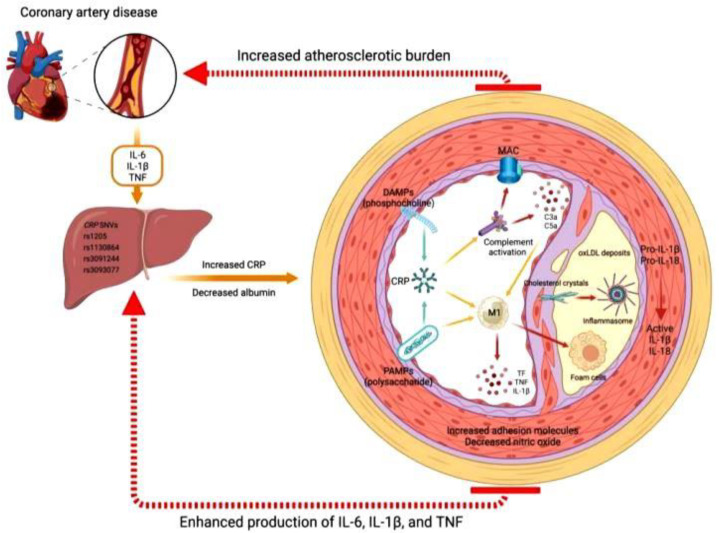
Mechanisms of CRP in atherosclerotic CVD. CRP, synthesized in the liver in response to IL-6, IL-1β, and TNF, binds to DAMPs and PAMPs, activating the complement cascade and macrophages. This inflammatory response promotes oxidized LDL deposition, foam cell formation, and inflammasome activation, increasing IL-1β and IL-18 production. The resulting endothelial dysfunction and chronic inflammation contribute to an increased atherosclerotic burden and the progression of CAD. Figure is adapted from Amezcua-Castillo E et al. [24].

CRP, primarily synthesized in the liver in response to proinflammatory cytokines like IL-6, IL-1β, and TNF, plays a crucial role in the pathogenesis of atherosclerotic cardiovascular disease (ASCVD). Genetic variations in CRP can modulate its circulating levels, influencing disease progression. CRP binds to danger-associated molecular patterns (DAMPs), such as phosphocholine from apoptotic debris; and pathogen-associated molecular patterns (PAMPs), like bacterial lipopolysaccharides. Upon activation, CRP undergoes a structural change, triggering the complement cascade and macrophage activation (M1 phenotype). This inflammatory response enhances the deposition of oxidized LDL (oxLDL) and foam cells in the vascular sub-endothelium, promoting atherogenesis. Cholesterol crystals in atheromatous plaques further activate inflammasomes, increasing IL-1β and IL-18 production. This leads to heightened expression of adhesion molecules, decreased nitric oxide levels, and vascular dysfunction. The chronic inflammatory state exacerbates the atherosclerotic burden, ultimately contributing to the progression of CAD (Figure 3) [24]. CRP serves as an independent predictor of future cardiac events, including myocardial infarction. Its early appearance in the bloodstream during systemic inflammatory disorders underscores its diagnostic significance in assessing cardiovascular risk, providing valuable prognostic information beyond traditional risk factors. One of the most promising forms is high-sensitivity C-reactive protein (hs-CRP), a biomarker of inflammation that has consistently been shown to predict incident myocardial infarction, stroke, and cardiovascular death among apparently healthy men and women after adjustment for all components of the Framingham risk score. Blood levels of CRP also correlate with hypofibrinolysis and abnormal glucose metabolism, reflecting pathophysiologic processes related to vascular occlusion that are not easily measured with traditional risk factors [25]. In the context of CAD, CRP’s ability to bind to DAMPs and PAMPs leads to the activation of the complement cascade and inflammatory macrophages. This contributes to the chronic inflammatory state within the vascular walls, facilitating the deposition of oxidized LDL and foam cells, which are key components of atherosclerotic plaques. Hence, CRP serves as an important biomarker for assessing cardiovascular risk, offering insights into the underlying inflammatory processes involved in CAD development and progression [26].

### 4.7. Myeloperoxidase (MPO)

MPO is an enzyme secreted by neutrophils and macrophages during inflammation, serving as a critical risk marker for acute coronary syndrome and chest discomfort by reflecting oxidative stress and inflammation [27]. Elevated MPO levels are associated with an increased risk of cardiovascular disease, as they contribute to endothelial dysfunction and plaque instability, making MPO a vital biomarker in all phases of endothelial pathology. MPO’s role extends to promoting lipid peroxidation, impairing nitric oxide bioavailability, and enhancing the formation of foam cells, all of which are pivotal in the initiation and progression of atherosclerosis. Thus, MPO not only signals existing cardiovascular risks but also highlights ongoing inflammatory processes that may lead to adverse cardiac events [28].

### 4.8. Cardiac Troponins

Cardiac troponin I (cTnI) is a highly specific marker for myocardial damage, making its elevation in the serum during myocardial ischemia symptomatology crucial for the diagnosis of myocardial infarction (MI). Among the various isoforms, cardiac troponin I (cTnI) and cardiac troponin T (cTnT) are considered the most specific markers for acute coronary syndromes (ACSs), and their elevations have become predominant indicators for acute myocardial infarction (AMI). Cardiac troponins are widely recognized as the gold-standard biomarkers for diagnosing AMI due to their high sensitivity and specificity. However, various chronic cardiovascular conditions can also lead to persistently elevated cardiac troponin levels, complicating their interpretation in clinical practice. The diverse pathophysiological mechanisms contributing to this elevation include direct myocardial injury, excessive wall stress, reduced oxygen supply, and increased myocardial oxygen demand. These mechanisms are commonly observed in conditions such as heart failure (HF), systemic hypertension, aortic stenosis, chronic kidney disease (CKD), and diabetes mellitus, highlighting the need for a comprehensive clinical assessment when interpreting troponin levels in chronic disease settings [29]. The rationale behind the cardiac troponin assay is straightforward: myocardial necrosis leads to membrane disruption, causing the release of troponins, which can then be detected in the serum. The use of troponins to diagnose acute myocardial injury has become a cornerstone of the Universal Definition of Acute Myocardial Infarction. However, with the advent of high-sensitivity cardiac troponin (cTn) assays, it has become evident that cTn is detectable in virtually everyone and can be elevated above the 99th percentile in stable chronic conditions, complicating its interpretation. While the release of troponins into the bloodstream following myocardial injury makes them invaluable for diagnosing heart conditions, their extended half-life poses challenges in identifying reinfarction events, necessitating complementary diagnostic approaches [30].

### 4.9. Hydroxybutyrate Dehydrogenase (HBDH)

HBDH, specifically α-hydroxybutyrate dehydrogenase, is an important enzyme whose activity determination is utilized in diagnosing myocardial infarction. Raised serum levels of HBDH are often observed shortly after a heart attack, reflecting myocardial damage. This enzyme is a marker of cell death, particularly indicating renal, red blood cell, and myocardial injury [31]. HBDH is notably associated with atherothrombotic events in patients undergoing infrainguinal angioplasty and stenting, and it may represent a more sensitive risk marker for these outcomes compared to traditional laboratory values. Elevated HBDH levels can indicate ischemic outcomes following such procedures for stable peripheral artery disease (PAD). Thus, HBDH is not only valuable for its diagnostic capabilities in acute myocardial infarction but also as a prognostic marker in chronic or acute ischemic heart disease and cerebrovascular disease. The prognostic value of HBDH extends to its association with the occurrence of atherothrombotic events after infrainguinal angioplasty with stent implantation. Its levels in the blood provide insight into the extent of cellular damage and the risk of further ischemic events, making it a critical biomarker in the management of CVDs [32].

### 4.10. Matrix Metalloproteinases (MMPs) and Tissue Inhibitors of Metalloproteinases (TIMPs)

**Figure 4:**
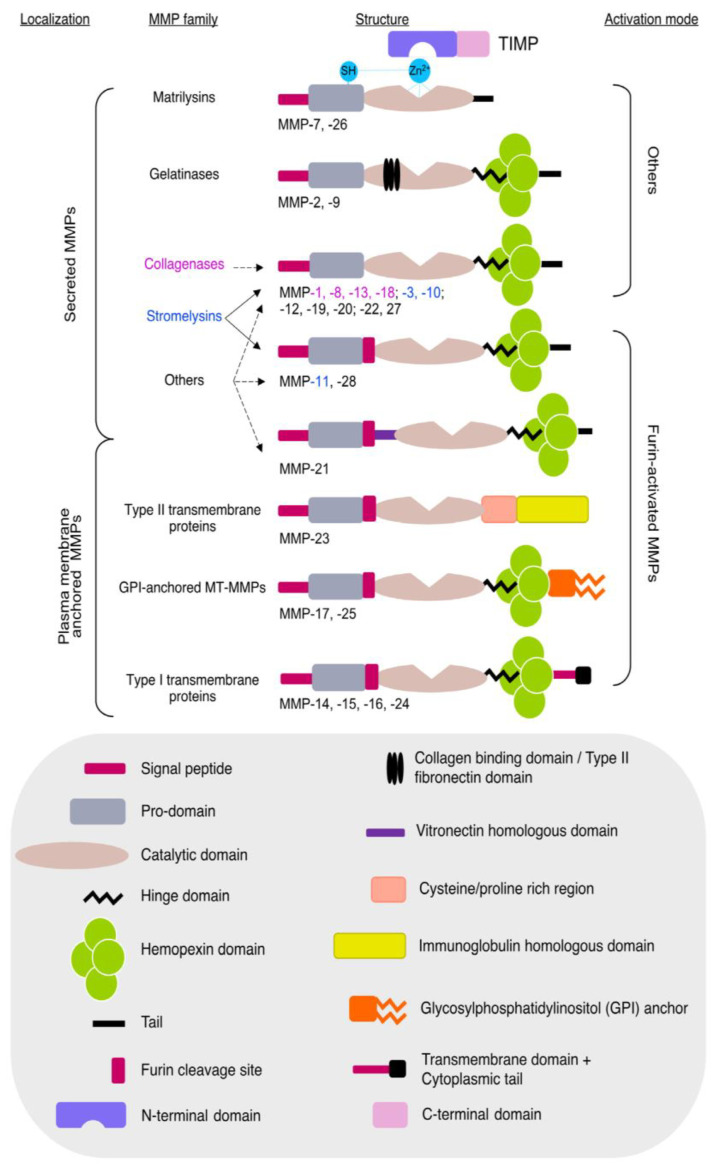
Diagram illustrating the structural domains of MMPs and TIMPs, categorized by sequence similarity and cellular location. Secreted MMPs include matrilysins, gelatinases, stromelysins, and collagenases, whereas MT-MMPs and GPI-anchored proteinases remain membrane-bound. The gray box highlights the specific domains of MMPs and TIMPs. Figure is adapted from Molière et al. [33].

Matrix metalloproteinases (MMPs) are calcium-dependent, zinc-containing endopeptidases that break down the extracellular matrix (ECM), facilitating tissue remodeling both during development and in adulthood. This enzyme family consists of 26 members encoded by 24 genes, categorized into collagenases, gelatinases, stromelysins, matrilysins, membrane-type MMPs, and others based on their function and localization (Figure 4). Their activity is tightly regulated by endogenous inhibitors, known as tissue inhibitors of metalloproteinases (TIMPs). Dysregulation of MMPs is implicated in various diseases, including cardiovascular disorders and cancer [33]. MMPs and TIMPs are implicated in cardiac extracellular matrix remodeling, play a pivotal role in ventricular remodeling and fibrosis, impacting heart failure prognosis. MMP-2, MMP-3, MMP-9, and TIMP-1 levels offer valuable insights into mortality risk and adverse outcomes in heart failure patients. MMPs are crucial in the development and progression of atherosclerosis and other CVDs, with their alteration being linked to increased cardiovascular morbidity and mortality. In CVDs, there are changes in the degradation and regeneration of the extracellular matrix (ECM) due to vascular wall instability secondary to disease-related damage. MMPs, particularly MMP-2, can degrade myofilament proteins related to contractility, reducing the Ca2+ sensitivity of myofilaments and leading to contractile dysfunction, as observed in ischemia–reperfusion scenarios [34]. Current research highlights the pathological roles of intracellular MMPs, especially MMP-2, which is abundant and actively retained within the cytoplasm. MMP-2 is involved in cardiac injury and repair, can impair ventricular function in the absence of superimposed injury, and may play roles in the maintenance of sarcomere proteostasis within striated muscle [35]. MMPs are significant in various cardiovascular pathologies, including aneurysm formation, coronary artery disease, MI, atherosclerosis, arterial hypertension, and heart failure. TIMPs also play significant roles in tissue remodeling related to cardiac function. TIMP-2 inhibits bFGF-induced endothelial cell proliferation, TIMP-3 inhibits cell proliferation and migration of stimulated endothelial cells, and TIMP-4 inhibits endothelial cells. The therapeutic use of TIMP inhibition for vascular disease is currently under development. Given their involvement in ECM remodeling and cardiovascular pathology, MMPs and TIMPs serve as important biomarkers for cardiovascular diseases [36].

### 4.11. Fibrinogen Levels

Elevated fibrinogen levels are a robust biomarker for diagnosing and prognosticating cardiovascular diseases. Fibrinogen, a pivotal component in the coagulation cascade, exhibits acute phase response elevation in the presence of cytokines, serving as a significant cardiovascular risk marker independent of traditional risk factors [37]. Its association with genetic variants underscores its emerging role as a predictor of cardiac risk, though challenges persist in differentiating fibrinogen levels in severe hepatic illness. Additionally, elevated γ′ fibrinogen levels are linked to the formation of clots that are resistant to fibrinolysis, further increasing CVD risk. Understanding the genetic polymorphisms in fibrinogen genes (FGA, FGB, and FGG) and their interactions with other genetic and inflammatory markers is crucial in utilizing fibrinogen as a reliable diagnostic and prognostic tool in cardiovascular diseases [38].

### 4.12. Myoglobin

Myoglobin is an iron- and oxygen-binding protein abundantly present in the heart and skeletal muscle of animals. It is not found in any tissue other than muscle, but it can be present in the bloodstream as a result of muscle damage. Myoglobin is a sensitive marker for AMI due to its rapid release from the myocardium during injury and its quick excretion by the kidneys within 24 h. It rises within the first 30 min after the onset of an acute event, peaking at 4–12 h, and returns to baseline values within 24–36 h [39,40]. However, myoglobin lacks specificity to cardiac tissue, as it is also abundant in skeletal muscle. Consequently, while elevated myoglobin levels can indicate muscle damage, they are not specific to cardiac injury. Therefore, myoglobin levels are used to rule out, rather than diagnose, myocardial infarction, and are often measured in conjunction with more specific cardiac markers, such as troponins or CK-MB, to enhance diagnostic accuracy. This underscores the importance of multimodal diagnostic approaches in clinical practice for the early detection and management of myocardial infarction [41].

### 4.13. Ischemia-Modified Albumin (IMA)

IMA is an emerging cardiac biomarker, arising from the interaction of serum albumin with ischemic cardiac tissue. IMA is characterized by its inability to bind cobalt ions, a feature exploited in the albumin cobalt-binding test. This test, which has received FDA approval, is a rapid diagnostic tool for myocardial ischemia with a laboratory turnaround time of approximately 30 min. IMA levels increase immediately after the onset of ischemia and return to baseline within 6–12 h, enabling early detection of ischemia before myocardial necrosis occurs. This makes IMA a valuable adjunctive biomarker in acute cardiovascular care, capable of distinguishing between ischemic and non-ischemic patients [42,43]. The albumin cobalt binding test, which detects structural changes in the N-terminus of albumin, shows a sensitivity of 70%, specificity of 80%, and a positive predictive value of 96% for acute coronary syndrome. However, the specificity of IMA is limited by its elevation in conditions other than myocardial ischemia, such as cancer, infection, brain ischemia, liver disease, and end-stage renal disease. Despite this, IMA remains the only clinical biomarker specifically for myocardial ischemia, offering a promising tool for rapid diagnosis and early intervention in acute cardiovascular events [44].

### 4.14. Glycogen Phosphorylase Isoenzyme BB (GPBB)

GPBB is a critical enzyme in carbohydrate metabolism, specifically involved in glycogen mobilization. Predominantly expressed in the brain and heart, GPBB plays a pivotal role in glycogenolysis, catalyzing the conversion of glycogen into glucose-1-phosphate during metabolic stress. Under normal physiological conditions, GPBB is tightly bound to the sarcoplasmic reticulum (SR) within cardiac cells. However, during ischemic events, GPBB is released into the cytosol and subsequently into the bloodstream. The ischemic release of GPBB is driven by the activation of glycogen phosphorylase, where ischemia promotes the conversion of GPb (non-phosphorylated, AMP-dependent form) to GPa (phosphorylated, active form). This transition accelerates glycogen breakdown, generating glucose-1-phosphate (G-1-P) and enabling GPBB to transition into a soluble form. The efflux of GPBB into the extracellular space occurs primarily when ischemia-induced structural damage compromises the integrity of the cell membrane [45]. GPBB’s clinical significance lies in its diagnostic specificity for myocardial injury when brain damage is excluded, as it is not present in other tissues under normal conditions. Its utility as a biomarker for AMI was first demonstrated over 25 years ago, with studies revealing its rapid release into circulation during the early phases of ischemic events. Subsequent research has reinforced these findings, confirming GPBB’s sensitivity and specificity in diagnosing ischemic heart disease, particularly in the early hours following symptom onset. Notably, GPBB is invaluable for detecting myocardial hypoxia in conditions such as carbon monoxide poisoning, where conventional symptoms or electrocardiogram (ECG) changes may be absent. By catalyzing the initial rate-limiting step in glycogenolysis, GPBB underscores the heart’s metabolic response to ischemia. Although its diagnostic specificity can be affected by elevations in other pathological states, such as pregnancy or brain injury, GPBB remains a crucial secondary biomarker when used alongside other cardiac markers, like troponins or myoglobin. In clinical practice, the early release of GPBB into the bloodstream enhances the rapid diagnosis and timely management of AMI, ultimately improving patient outcomes in acute cardiovascular care. Its utility, particularly in the early hours of ischemia, highlights its role as an essential tool in the diagnostic arsenal for acute coronary syndromes [46,47].

### 4.15. Oxylipins

Oxylipins derived from arachidonic acid (AA) include epoxyeicosatrienoic acids (EETs), hydroxyeicosatetraenoic acids (HETEs), and prostanoids, while those originating from linoleic acid (LA) encompass epoxyoctadecenoic acids (EpOMEs), hydroxyoctadecadienoic acids (HODEs), and others. Various oxylipins synthesized from AA and LA, along with the primary enzymes responsible for their production and degradation include lipoxygenases, cytochrome P450 epoxygenases, ω-hydroxylases, and cyclooxygenases [48]. Key oxylipins, such as epoxyeicosatrienoic acids (EETs) and dihydroxyeicosatrienoic acids (DHETs), play critical roles in vasodilation and anti-inflammatory responses. EETs have been noted for their protective effects against ischemia/reperfusion injury, whereas DHETs, primarily generated by soluble epoxide hydrolase (sEH), can exert less favorable, pro-inflammatory actions [48,49]. Genetic variations in enzymes involved in oxylipin metabolism, such as cytochrome P450 epoxygenases (CYP2J2) and sEH, significantly influence cardiovascular disease susceptibility. Plasma levels of EETs; DHETs; and other oxylipins, like hydroxyeicosatetraenoic acids (HETEs) and prostanoids (e.g., PGF2α and PGE2), have shown correlations with cardiac function and inflammatory states, making them promising noninvasive indicators for diagnosing and monitoring cardiovascular conditions. By understanding the nuanced roles of oxylipins in cardiovascular physiology and pathology, researchers aim to uncover novel biomarkers and therapeutic targets, potentially revolutionizing the approach to cardiovascular disease management [50].

### 4.16. Lipoprotein-Associated Phospholipase A2 (Lp-PLA2)

Lp-PLA2 has emerged as a pivotal biomarker in CVD, providing valuable insights into the pathophysiology, diagnostic, and prognostic implications of vascular inflammation and atherosclerosis (Table 1). As a member of the phospholipase A2 superfamily, Lp-PLA2 is an enzyme predominantly synthesized by inflammatory cells within atherosclerotic plaques. Its primary role involves the hydrolysis of phospholipids, which is crucial in the context of lipid metabolism and inflammatory response within the vascular system [51]. Notably, Lp-PLA2 exhibits a significant predilection for binding with specific lipoprotein fractions, including high-density lipoprotein (HDL), low-density lipoprotein (LDL), and very low-density lipoprotein (VLDL). This affinity underscores its atherogenic potential, as these lipoprotein complexes are intricately linked to the development and progression of atherosclerotic lesions. The enzyme’s initial identification as plasma platelet-activating factor acetylhydrolase (pPAF-AH) highlights its hydrolytic function on platelet-activating factor (PAF), a potent proinflammatory mediator. Inflammation is a cornerstone in the pathogenesis of vascular diseases, with Lp-PLA2 playing a critical role in mediating vascular inflammation through its regulatory effects on lipid metabolism. Elevated levels of Lp-PLA2 have been consistently associated with an increased risk of various vascular diseases, particularly atherosclerosis. This enzyme’s involvement in vascular inflammation underscores its utility as a biomarker for assessing predisposition to future cardiovascular events [52]. The diagnostic utility of Lp-PLA2 is further bolstered by its high specificity for vascular inflammation and minimal biological variability. This makes it an ideal marker for CVDs, providing nuanced information regarding vascular inflammation that is therapeutically relevant. Elevated Lp-PLA2 levels have been correlated with an increased risk of cardiovascular events, including CHD and ischemic stroke. Lp-PLA2 serves not only as a standalone risk factor for CVD but also as a potential therapeutic target. By reflecting the inflammatory state within atherosclerotic plaques, Lp-PLA2 levels offer a prognostic indicator for cardiovascular risk, enabling more personalized and targeted interventions. The enzyme’s proinflammatory and lipid regulatory roles are critical in the context of atherosclerosis, making it an invaluable biomarker for predicting and managing cardiovascular complications. Hence, Lp-PLA2’s role as a cardiac biomarker is underscored by its involvement in vascular inflammation, lipid metabolism, and atherogenesis [53]. Its high specificity and minimal biological variability make it a robust marker for assessing cardiovascular risk, particularly in individuals predisposed to vascular diseases. As research continues to elucidate its mechanisms, Lp-PLA2 stands poised as a cornerstone in the diagnosis, prognosis, and management of cardiovascular diseases.

### 4.17. B-Type Natriuretic Peptide (BNP) and N-Terminal Pro-B-Type Natriuretic Peptide (NT-proBNP)

BNP and NT-proBNP are crucial cardiac biomarkers widely used in the diagnosis, prognosis, and management of various CVDs [54]. These peptides are released in response to myocardial stretch and stress, making them sensitive indicators of cardiac dysfunction. Table 2 provides comparison of BNP and NT-proBNP, vital cardiac biomarkers used to diagnose, assess the prognosis of, and manage various cardiovascular diseases. In heart failure (HF), BNP and NT-proBNP levels rise significantly, correlating with the severity of the condition. These biomarkers are not only diagnostic but also prognostic, helping to assess the risk of future cardiovascular events and mortality. They have proven to be strong predictors for HF with preserved ejection fraction (HFpEF) and are used clinically to guide therapy and evaluate its effectiveness. Diastolic dysfunction is a prevalent yet diagnostically challenging condition compared to systolic dysfunction, often requiring advanced biomarkers for accurate detection. Natriuretic peptides, including BNP and NT-proBNP, play a crucial role in assessing ventricular filling pressures and myocardial stress. Additionally, soluble ST2, a marker of myocardial fibrosis and remodeling, has been shown to improve the echocardiographic diagnosis of elevated left ventricular filling pressures, offering better diagnostic precision. These biomarkers provide valuable insights into disease progression and may aid in the early identification of HFpEF [55]. Studies show a positive correlation between elevated BNP/NT-proBNP levels and the risk of death, highlighting their importance in patient management. BNP and NT-proBNP also play a significant role in ischemic heart diseases. Elevated levels are associated with AMI and acute coronary syndrome (ACS), reflecting the severity of left ventricular dysfunction. These biomarkers are effective in predicting adverse cardiovascular events and guiding aggressive treatments aimed at reducing ventricular wall stress. Their levels correlate with the size of myocardial infarction, making them valuable in prognosis and severity assessment [56]. In cases of arrhythmias and cardiomyopathies, BNP and NT-proBNP are elevated, indicating cardiac stress and dysfunction. For instance, patients with atrial fibrillation or ventricular arrhythmias show increased levels of these peptides. In cardiomyopathies, such as dilated or hypertrophic cardiomyopathy, BNP/NT-proBNP levels directly correlate with left ventricular end-diastolic dimension (LVEDD) and are inversely related to left ventricular ejection fraction (LVEF), providing insights into disease progression and severity. Furthermore, BNP and NT-proBNP have forensic applications in assessing end-stage cardiac function. Postmortem studies reveal elevated levels in individuals who died from acute ischemic heart disease, chronic congestive heart failure, and other cardiac conditions. These biomarkers do not fluctuate significantly after death, making them reliable indicators of premortem cardiac function. Elevated BNP in pericardial fluid and myocardial tissue helps distinguish cardiac-related deaths from other causes, aiding forensic investigations [57].

### 4.18. Mid-Regional Pro-Atrial Natriuretic Peptide (MR-proANP) and Mid-Regional Pro-Adrenomedullin (MR-proADM)

MR-proANP is a valuable biomarker for CVDs. Derived from the stable N-terminal portion of proANP, MR-proANP has a longer half-life than atrial natriuretic peptide (ANP), making it a reliable analyte for measurement. MR-proANP has shown significant promise in diagnosing and prognosing heart failure (HF). While its diagnostic utility was slightly lower than BNP and NT-proBNP, MR-proANP excelled in prognostic value, predicting mortality more accurately over five years [58]. In chronic HF, MR-proANP has shown robust prognostic capabilities, being significantly associated with mortality and proving crucial for monitoring disease progression and treatment efficacy. Beyond HF, MR-proANP holds potential in broader cardiovascular applications, such as screening for atrial fibrillation (AF) in community populations. Unlike NT-proBNP, MR-proANP effectively identifies individuals at risk of developing AF. Similar to BNP and NT-proBNP, MR-proANP levels are influenced by age, BMI, race, and sex. Despite these variations, MR-proANP’s stability and strong prognostic value make it an indispensable tool in the cardiac biomarker arsenal, particularly for personalized patient management in cardiovascular care [59].

MR-proADM has emerged as a potent cardiac biomarker with significant prognostic value in both acute and chronic heart failure (HF), as well as in acute coronary syndrome (ACS) and acute myocardial infarction (AMI). MR-proADM is a stable fragment of adrenomedullin, a peptide involved in vasodilation, natriuresis, and diuresis, which reflects the pathophysiological processes of HF and cardiovascular stress. In patients with ACS and AMI, MR-proADM levels are independently associated with both fatal and nonfatal cardiovascular events [60]. The AtheroGene study and Leicester Acute Myocardial Infarction Peptide (LAMP) study by khan et al. have demonstrated that MR-proADM adds prognostic information beyond traditional risk models and biomarkers like BNP and NT-proBNP. It enhances risk stratification, predicting mortality and heart failure more effectively than natriuretic peptides alone. This biomarker provides valuable prognostic insights independent of other factors, such as renal function, age, and systolic blood pressure. For chronic HF, MR-proADM’s levels correlate strongly with disease severity and NYHA class, offering predictive value for long-term outcomes. It has been shown to outperform BNP and NT-proBNP in predicting mortality and adverse events, particularly in patients with mild-to-moderate symptoms [61]. Table 3 highlights the complementary roles of MR-proANP and MR-proADM in cardiovascular care. MR-proANP excels in long-term HF management and AF screening, while MR-proADM provides superior prognostic insights in HF, ACS, and AMI, enhancing risk stratification and guiding clinical decision-making.

### 4.19. Endothelin-1 (ET-1)

**Figure 5:**
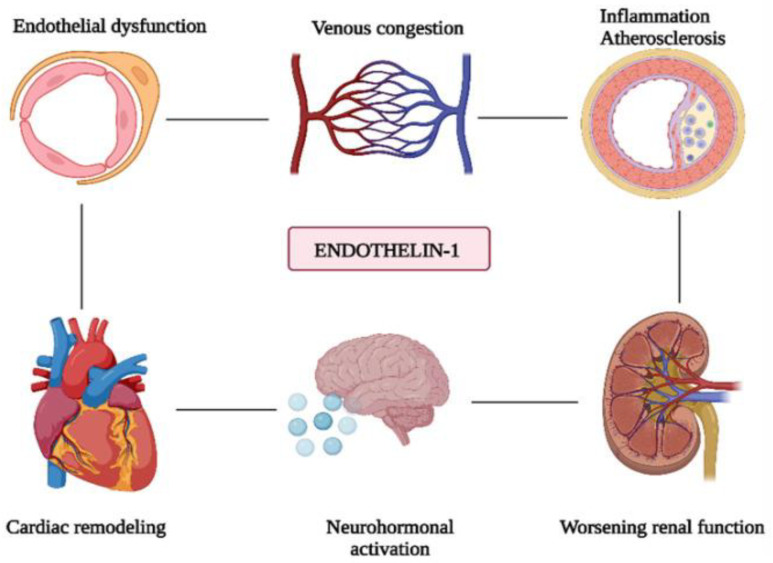
The role of ET-1 in the pathogenesis of AHF. ET-1 induces systemic and pulmonary vasoconstriction, promotes cardiac remodeling and neurohormonal activation, and worsens renal function, thereby contributing to vascular and cardiac dysfunction. Figure is adapted from Dmour et al. [62].

Endothelin-1 (ET-1) is a potent vasoconstrictor peptide produced by endothelial cells, playing a pivotal role in cardiovascular health and disease. It contributes significantly to the pathogenesis of acute heart failure (AHF) by inducing systemic, pulmonary, coronary, and renal vasoconstriction, leading to increased vascular resistance, elevated left ventricular filling pressures, and impaired renal function. ET-1 promotes sodium and fluid retention, exacerbating venous congestion and renal dysfunction. At the cardiac level, ET-1 is involved in myocardial ischemia and ventricular remodeling, directly stimulating myocardial hypertrophy and fibrosis. Elevated ET-1 levels are strongly associated with the severity and mortality of heart failure, making it a critical biomarker for disease progression (Figure 5) [62,63,64]. ET-1 exerts its effects through two G protein-coupled receptors: endothelin receptor A (ETA) and endothelin receptor B (ETB). ETA primarily mediates arterial vasoconstriction, while ETB can induce either vasoconstriction or vasodilation depending on its location. The peptide is synthesized via the conversion of its precursor, big ET-1, by endothelin-converting enzyme or intermediate metabolites that also lead to active ET-1 production. ET-1 plays a central role in endothelial dysfunction by tipping the balance between vasodilation and vasoconstriction toward the latter. This disruption leads to inflammation, thrombosis, and atherosclerotic lesion formation. It markedly reduces endothelium-dependent vasodilation, a vital vascular function assessed through noninvasive tests, like flow-mediated dilation (FMD) and reactive hyperemia (RH). Therapeutically, endothelin receptor antagonists that block ET-1’s actions have shown success in treating pulmonary arterial hypertension and are under investigation for resistant hypertension and chronic kidney disease. Emerging treatments targeting the endothelin pathway include small-molecule modulators, biologics like monoclonal antibodies, and pathway-specific agonists/antagonists. Beyond vasoconstriction, ET-1 contributes to vascular inflammation, cardiac hypertrophy, and disease progression in hypertension, chronic kidney disease, and atherosclerosis (Figure 5) [62,63,64].

### 4.20. Tumor Necrosis Factor-Alpha (TNFα)

Tumor necrosis factor-alpha (TNFα) is a pro-inflammatory cytokine predominantly produced by monocytes and macrophages, playing a critical role in the pathophysiology of cardiovascular diseases (CVDs). Elevated serum TNFα levels are commonly observed in heart failure (HF) patients across a spectrum of ejection fractions (EFs). These elevated levels are strongly correlated with decreased renal function, anemia, and an increased burden of comorbidities. Importantly, TNFα has been independently associated with increased mortality in HF patients regardless of EF, enhancing risk prediction beyond conventional markers [65].

TNFα exerts its effects through complex mechanisms. It activates multiple signaling pathways, kinases, and transcription factors by binding to specific cell surface receptors, disrupting normal intracellular calcium dynamics. Initially identified as a contributor to cardiac dysfunction in sepsis, TNFα has since been implicated in several cardiac conditions, including myocarditis, hypertrophic cardiomyopathy, myocardial infarction, and post-cardiopulmonary bypass dysfunction. In animal studies, increased cardiac expression of TNFα in failing myocardium leads to left ventricular dysfunction, dilation, adverse remodeling, and hypertrophy [66]. Despite its deleterious effects, TNFα may offer some protective benefits, particularly in acute ischemic injury. However, its overall role in HF is complex and remains incompletely understood.

In HF patients with preserved EF (HFpEF), TNFα levels have been found to be elevated in about one-third of cases. These elevations are associated with significant comorbidities and reduced renal function. The inflammatory state of HF, marked by elevated TNFα and other cytokines, arises in response to various cardiac stresses, such as hypertension, diabetes, and ischemic heart disease. While acute TNFα-mediated inflammation may support tissue repair and remodeling, chronic inflammation caused by sustained cardiac stress leads to persistently high TNFα levels, promoting adverse remodeling and negative inotropic effects. This prolonged inflammatory state is further exacerbated by macrophage infiltration into cardiac tissue, perpetuating the release of pro-inflammatory cytokines [67]. The impact of TNFα extends to endothelial cells, smooth muscle cells, and cardiac myocytes. In endothelial cells, TNFα induces inflammatory gene expression, leukocyte recruitment, cytoskeletal reorganization, and apoptosis. In smooth muscle cells, it promotes proliferation, migration, and constriction while triggering inflammatory gene expression and apoptosis. In cardiac myocytes, TNFα contributes to decreased contractility, hypertrophy, inflammatory gene expression, and apoptosis [65,66,67,68].

### 4.21. Interleukin-6 (IL-6)

**Figure 6:**
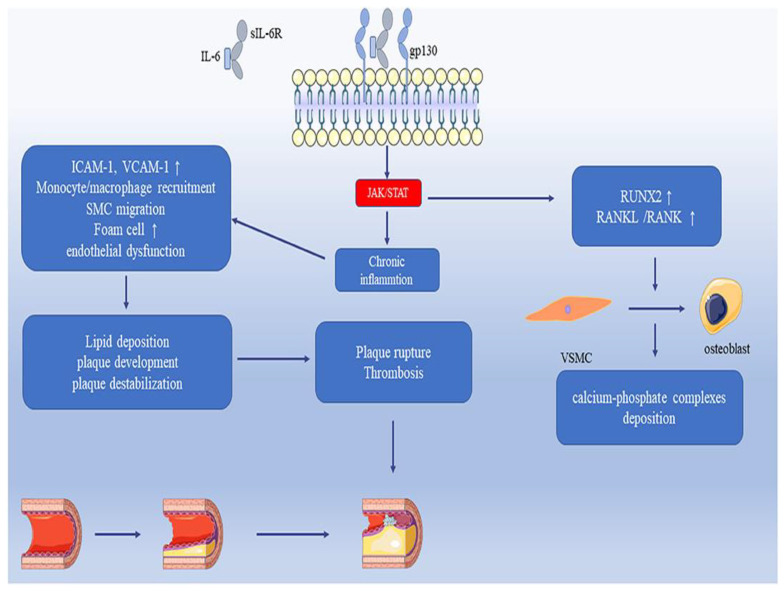
Demonstrates the role of IL-6 in atherosclerosis, myocardial infarction, and vascular calcification. IL-6 trans-signaling activates the JAK/STAT pathway, inducing chronic inflammation, endothelial dysfunction, and lipid deposition, which drive plaque formation and rupture. IL-6 also upregulates RUNX2 and RANKL, promoting vascular smooth muscle cell differentiation into osteoblasts, contributing to vascular calcification. Figure is adapted from Feng et al. [72].

IL-6 is a significant biomarker in the context of CVDs, playing a crucial role in the inflammatory response, vascular inflammation, and atherosclerosis. As an acute-phase protein, IL-6 contributes to the remodeling of connective tissue by increasing metalloproteinase gene expression. This process can destabilize atherosclerotic plaques, leading to plaque instability, a critical factor in the progression of CAD. IL-6 levels are strongly associated with mortality in patients with unstable CAD, independent of traditional risk factors such as age, sex, diabetes, and previous myocardial infarction (MI) [69]. Elevated IL-6 levels predict cardiovascular events in both unstable and stable coronary disease, underscoring its utility as a biomarker for adverse cardiovascular outcomes. Studies have shown that high IL-6 concentrations are predictive of major adverse cardiovascular events, including myocardial infarction, heart failure, and mortality [70]. In atherosclerosis, IL-6 promotes vascular inflammation by encouraging smooth muscle cell proliferation, endothelial dysfunction, and the activation of inflammatory mediators. These processes contribute to the development and destabilization of atherosclerotic plaques. Higher IL-6 levels, particularly following ST-elevation myocardial infarction (STEMI), are linked to larger infarct sizes and reduced cardiac function, making IL-6 a potential biomarker for STEMI prognosis. Moreover, IL-6 plays a role in the chronic inflammatory process associated with CVDs [71]. Chronic administration of IL-6 antagonists, such as tocilizumab and canakinumab, has shown potential in reducing inflammatory responses and major adverse cardiovascular events, highlighting IL-6’s role as a therapeutic target. IL-6 promotes early atherosclerosis by driving chronic inflammation and immune cell recruitment, but its lifelong deficiency exacerbates the disease, highlighting its complex effects. Through JAK/STAT signaling, IL-6 regulates vascular inflammation, endothelial dysfunction, and smooth muscle cell migration, contributing to plaque formation, destabilization, and thrombosis. Additionally, IL-6 upregulates RUNX2 and RANKL/RANK pathways, inducing vascular smooth muscle cell differentiation into osteoblast-like cells, leading to vascular calcification (Figure 6) [72].

### 4.22. Growth Differentiation Factor-15 (GDF-15)

GDF-15 has emerged as a key biomarker in heart failure (HF) and other cardiovascular conditions, reflecting its involvement in incidence, diagnosis, progression, and prognosis. Elevated GDF-15 levels are associated with an increased risk of developing HF, including post-myocardial infarction HF [73,74,75,76]. In diagnosis, GDF-15 enhances the detection accuracy of HF with preserved ejection fraction (HFpEF) [75]. Regarding disease progression, GDF-15 correlates with cardiac remodeling and worsening New York Heart Association (NYHA) functional class [75]. Prognostically, GDF-15 predicts rehospitalization for HF and is strongly linked to mortality in HF patients, irrespective of ejection fraction status [75]. Elevated levels are also associated with sudden death in patients with reduced ejection fraction (HFrEF) and stable coronary artery disease (CAD) [76]. Beyond HF, GDF-15 plays a role in inflammation-driven cardiovascular conditions, such as atherosclerosis, by mitigating foam cell formation and suppressing lipid accumulation [74]. In acute coronary syndrome (ACS), GDF-15 predicts recurrence of myocardial infarction and cardiac death. When used alongside biomarkers like NT-proBNP, it improves risk stratification in high-risk groups [75]. Additionally, GDF-15 serves as a sensitive and specific marker for diagnosing cardiac amyloidosis and assessing the severity of peripheral artery disease (PAD) [76].

### 4.23. Suppression of Tumorigenicity-2 (ST2)

ST2, a member of the interleukin-1 receptor family, has two main forms: the transmembrane ST2L and the soluble sST2. The ligand for ST2 is interleukin-33 (IL-33), which binds to ST2L and triggers signaling pathways with immunomodulatory effects across various cell types, including those involved in tumor, immune, and cardiac functions. Soluble ST2 (sST2), released into the circulation, acts as a decoy receptor for IL-33, inhibiting IL-33/ST2L signaling and its beneficial effects. The ST2/IL-33 axis, particularly the role of sST2, has been implicated in several inflammatory, cardiac, and cancer-related pathologies. sST2 is notably involved in the pathogenesis and homeostasis of CVDs, where it serves as a counterbalance to the IL-33/ST2L axis. This balance is critical in conditions involving fibrosis, tissue damage, inflammation, and remodeling, all of which are common in CVDs [77]. Elevated sST2 levels reflect these pathophysiological changes and have been recognized as a valuable prognostic biomarker in various cardiac conditions, including heart failure and coronary artery disease. In acute heart failure, studies have shown higher sST2 levels in non-survivors compared to survivors. Although there is substantial variability in findings, elevated sST2 levels have been correlated with an increased risk of all-cause mortality. This suggests that sST2 can provide important prognostic information, but further research is needed to conclusively determine its value in acute settings [78,79]. In chronic heart failure, elevated sST2 levels have been significantly associated with higher mortality rates. Studies have demonstrated that higher sST2 levels correlate with worse outcomes, making it a robust prognostic marker. This relationship underscores the potential of sST2 to reflect disease severity and predict adverse outcomes in chronic heart failure patients [80]. In stable coronary artery disease, elevated sST2 levels are consistently associated with increased mortality. This association suggests that sST2 can be a useful tool for risk stratification and prognosis in patients with stable coronary artery disease, aiding in identifying those at higher risk of adverse outcomes [81]. The consistent correlation of elevated sST2 levels with increased mortality and disease severity across various cardiovascular conditions highlights sST2 as a promising biomarker for assessing prognosis and guiding treatment decisions. Its role in reflecting underlying pathophysiological processes, such as fibrosis, inflammation, and cardiac remodeling, makes it a valuable tool for clinicians. However, accurate measurement and interpretation of sST2 levels are crucial, necessitating standardized methods and recognition of factors influencing assay results.

### 4.24. Pentraxin 3 (PTX3)

PTX3 is an evolutionarily conserved, multimeric acute phase inflammatory glycoprotein, belonging to the same family as the well-established cardiovascular biomarker C-reactive protein (CRP). Identified through differential screening and proteomic approaches in human endothelial cells, PTX3 is primarily synthesized in response to inflammatory stimuli, such as cytokines and endotoxins. It plays a significant role in CVDs by regulating inflammation, oxidative stress, and vascular endothelial function [82]. PTX3 expression is notably elevated in patients with acute myocardial infarction (AMI). Studies have shown that PTX3 levels peak shortly after the onset of AMI and return to baseline within a few days. In patients with unstable angina pectoris (UAP), increased PTX3 levels have been observed, suggesting its potential as a prognostic marker. Elevated PTX3 levels have been linked to higher risks of adverse cardiac events and mortality in ACS patients. In patients with heart failure, PTX3 has emerged as a predictor of adverse clinical outcomes. Increased PTX3 levels correlate with disease severity and poor prognosis. This is true even in patients with heart failure with normal ejection fraction (HFNEF), where PTX3 levels are elevated despite normal levels of other markers, like B-type natriuretic peptide (BNP). In conditions such as aortic valve stenosis (AS) or regurgitation (AR), PTX3 levels are significantly increased [83]. PTX3 is predominantly expressed in macrophage cells within the aortic valves, highlighting its role in the inflammatory processes associated with valvular heart disease. PTX3 plays a protective role against cardiac tissue damage, particularly in the context of AMI. Research using PTX3-knockout mice has demonstrated that the absence of PTX3 exacerbates heart damage and inflammatory response, whereas the introduction of exogenous PTX3 reverses these effects [84]. This protective role is also evident in atherosclerosis, where PTX3 reduces neutrophil adhesion and inflammation, thereby mitigating the progression of the disease. In models of sepsis and systemic inflammation, PTX3 levels rise significantly, indicating its broader role in inflammatory regulation beyond CVDs. Its anti-inflammatory properties are partly mediated through interactions with P-selectin, reducing neutrophil recruitment and adhesion, which are critical processes in the pathogenesis of atherosclerosis and other inflammatory conditions. Recent findings suggest that PTX3 is released from neutrophils by platelets in patients with acute coronary syndrome (ACS), particularly in the early stages of AMI. This release mechanism underscores PTX3’s role in modulating inflammation and protecting cardiac tissues during acute ischemic events. PTX3 is a multifaceted biomarker with significant potential in the diagnosis, prognosis, and management of various cardiovascular diseases. Its role in reflecting and regulating inflammatory processes makes it a valuable tool in clinical practice. As research continues to elucidate its mechanisms and effects, PTX3 may become integral to the therapeutic and predictive strategies for managing cardiovascular conditions, offering insights into disease progression and treatment efficacy [85].

### 4.25. Pregnancy-Associated Plasma Protein-A (PAPP-A)

PAPP-A, a member of the metzincin metalloproteinase superfamily, enhances local insulin-like growth factor (IGF) bioavailability through the proteolytic cleavage of IGF-binding proteins (IGFBPs) [86]. Recent studies highlight the significance of PAPP-A in cardiovascular diseases (CVDs), particularly coronary artery disease (CAD), due to its role in promoting atherosclerosis and serving as a biomarker for diagnosis and prognosis. PAPP-A accelerates the development of atherosclerosis through mechanisms such as lipid accumulation, vascular inflammation, endothelial dysfunction, and vascular smooth muscle cell (VSMC) proliferation and migration, contributing to plaque stability and thrombus formation [87]. Elevated serum levels of PAPP-A correlate with markers of early atherosclerosis, making it a potential biomarker for detecting subclinical atherosclerosis in asymptomatic patients, aiding in early intervention and improved prognosis. PAPP-A levels are significantly higher in patients with unstable angina or acute myocardial infarction compared to those with stable angina and healthy controls, and elevated circulating PAPP-A levels are associated with a higher risk of cardiovascular events, making it a valuable prognostic biomarker for CAD. Monitoring PAPP-A levels can help identify individuals at high risk of cardiovascular events, guiding preventive and therapeutic strategies. Given its proatherogenic effects, reducing PAPP-A bioactivity could be an attractive therapeutic approach for treating atherosclerosis, with strategies such as blocking its proteolytic activity with stanniocalcin or microRNAs showing protective effects against atherosclerosis development. This therapeutic potential underscore the importance of further research into PAPP-A as a target for CVD treatment [88]. PAPP-A is a multifaceted biomarker with significant diagnostic and prognostic value in various cardiovascular diseases, playing a crucial role in promoting atherosclerosis through multiple pathological mechanisms and highlighting its potential as both a marker for disease presence and progression and a target for therapeutic intervention. As research continues, PAPP-A may become an integral part of cardiovascular disease management, aiding in early diagnosis, risk assessment, and the development of novel treatment strategies [89].

### 4.26. Copeptin

**Figure 7:**
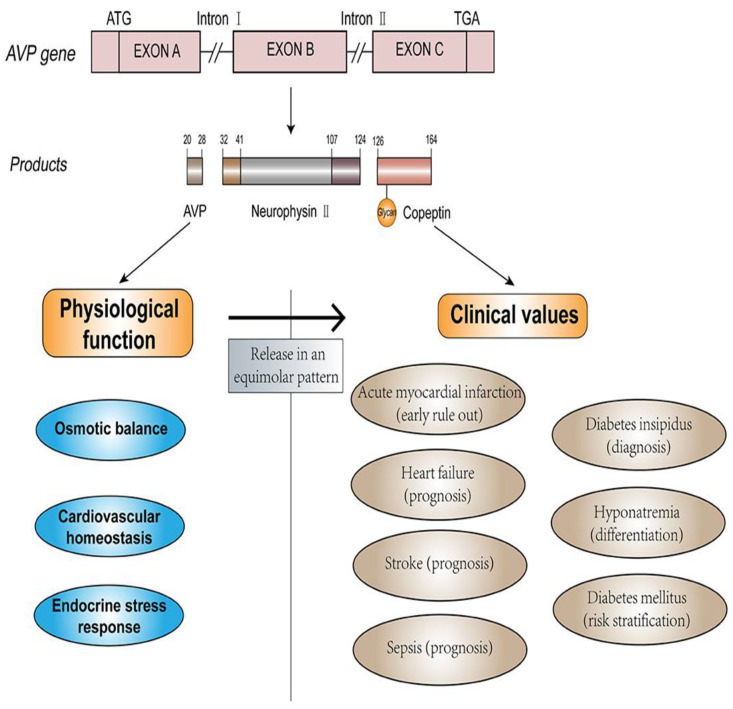
Structure, physiological functions, and clinical applications of AVP and copeptin were represented. Figure is adapted from Mu et al. [90].

Arginine vasopressin (AVP), also known as antidiuretic hormone, is a key regulator of fluid homeostasis, vasoconstriction, and the endocrine stress response, released in response to osmotic and hemodynamic stimuli. Copeptin, the C-terminal fragment of pro-AVP, is secreted in equimolar amounts with AVP and offers advantages as a stable and reliable biomarker. Its diagnostic potential has been widely evaluated, making it a valuable tool in clinical practice [90]. The structure, physiological function, and clinical values of copeptin and AVP were represented in Figure 7. Copeptin is derived from the cleavage of pre-pro-vasopressin, serves as a stable surrogate marker for vasopressin levels, making it a reliable indicator of fluid balance and severity in heart failure. Its utility is particularly evident in the diagnosis and prognosis of myocardial infarction (AMI). In the context of AMI, early detection and accurate diagnosis are crucial for prompt intervention. Traditional diagnostic methods, such as electrocardiography (ECG) and serum cardiac troponin T (cTnT), though essential, have limitations. ECG may not show significant changes in a substantial fraction of AMI patients, and conventional cardiac biomarkers like CK-MB and TnT do not rise in the initial hours of AMI onset, necessitating prolonged monitoring and serial blood sampling. Copeptin, released in response to acute endogenous stress, such as AMI, provides a significant advantage due to its rapid elevation within hours of chest-pain onset, peaking within the first day. This rapid increase makes copeptin a sensitive early marker of AMI when other biomarkers remain undetectable. Combining copeptin with cTnT enhances diagnostic accuracy, allowing for faster exclusion of AMI. Studies have shown that this combination can achieve high sensitivity and negative predictive value within hours of chest pain onset, significantly improving early diagnosis and management [91]. In heart failure, copeptin reflects the activation of the arginine vasopressin (AVP) system, a response to decreased cardiac output and other stressors. Elevated copeptin levels correlate with disease severity and prognosis, independently predicting poor outcomes in chronic heart failure patients. Copeptin, when combined with natriuretic peptides like BNP or NT-proBNP, provides a comprehensive assessment of heart failure by covering different pathological pathways. This combined measurement improves prognostic accuracy beyond what either biomarker could achieve alone. Moreover, copeptin levels are elevated post-ischemia and correlate with higher mortality risk and new-onset heart failure. Some studies suggest its superiority over BNP for predicting death, although the relationship between these biomarkers is closely intertwined. Copeptin’s role extends beyond AMI and heart failure to other acute and chronic cardiovascular conditions, underscoring its value as a general marker of physiological stress and disease severity. Its early rise and correlation with disease severity make it a crucial tool in the rapid and accurate assessment of cardiovascular diseases, enhancing clinical decision-making and patient management [92].

### 4.27. Galectin-3 (Gal-3)

Gal-3 has garnered significant attention due to its unique structure and function. Gal-3, a soluble β-galactoside-binding protein secreted by activated macrophages, is implicated in cardiac fibrosis, inflammation, and ventricular remodeling—key processes in the pathophysiology of HF. It binds to and activates fibroblasts, leading to collagen and scar tissue formation, which contributes to progressive cardiac fibrosis. Gal-3’s association with cardiac remodeling and HF development has been supported by numerous studies, which also highlight its potential as a prognostic marker [93]. Elevated Gal-3 levels are linked to higher risks of cardiovascular and all-cause mortality, as well as increased rates of complications in both acute and chronic HF. These findings apply to both heart failure with preserved ejection fraction (HFpEF) and heart failure with reduced ejection fraction (HFrEF) [94]. While some debate exists regarding whether Gal-3’s prognostic value is independent of renal and hepatic function parameters or incremental to other established biomarkers, like natriuretic peptides, its role in reflecting cardiac fibrosis and inflammation remains undisputed. Gal-3’s stability and resistance to hemodynamic changes offer advantages over other biomarkers. It serves as an early indicator of cardiac fibrosis, ventricular remodeling, and renal impairment in HF patients [95]. Combining Gal-3 with other biomarkers, such as NT-proBNP, could enhance prognostic accuracy and aid in the stratification of high-risk HF patients. Additionally, Gal-3’s biology and its role in HF pathology suggest potential therapeutic applications, warranting further research into its use as a target for treatment. Despite advances in HF treatment, including the use of ACE inhibitors, β-blockers, ARBs, MRAs, and implantable cardiac defibrillators, the prognosis for HF patients remains poor. Clinicians face challenges in risk prediction across acute, chronic, and new-onset HF. Biomarkers like Gal-3 can aid in risk stratification and guide the effective use of resources and therapies. Gal-3’s association with tissue fibrosis, a hallmark of cardiac remodeling, underscores its potential utility in HF diagnosis and management. The growing interest in Gal-3 as a biomarker reflects its significant role in cardiac pathology. Its elevation in chronic HF, regardless of etiology, and its predictive value in mortality highlight its importance. Combining imaging and biochemical biomarkers, such as Gal-3 with echocardiographic and CMR biomarkers, could improve diagnostic and prognostic accuracy. The development of Gal-3 as a routine test alongside established biomarkers like BNP and NT-proBNP could significantly enhance HF management and patient outcomes [96].

### 4.28. Trimethylamine n-Oxide (TMAO)

TMAO emerges as a biomarker for heart and kidney disease, generated from gut microbiota-mediated l-carnitine metabolism. TMAO has emerged as a significant biomarker in cardiometabolic health, drawing increasing attention for its association with CVDs. The link between TMAO and CVD has been extensively studied [97]. Using metabolomics, researchers have identified TMAO as part of a cluster of molecules, including choline and betaine, that are associated with atherosclerosis. In murine models of atherosclerosis, plasma TMAO levels correlate with atheroma burden, and dietary choline increases foam cell formation and the expression of scavenger receptors in macrophages. In clinical settings, elevated plasma levels of choline and betaine, particularly when accompanied by high TMAO levels, are associated with poor prognosis and higher risks of major cardiac events [98]. However, not all studies consistently demonstrate an association between TMAO and atherosclerotic vascular disease. For instance, some studies have found higher plasma TMAO levels in diabetics compared to non-diabetics, but no association with myocardial infarction or coronary heart disease. Additionally, studies on patients with large-artery atherosclerotic ischemic stroke and transient ischemic attack show significant gut microbiota dysbiosis and decreased blood TMAO levels, complicating the understanding of TMAO’s role in these conditions. Despite these mixed findings, substantial evidence supports the association between elevated TMAO levels and adverse cardiovascular events. Interestingly, seafood, which contains high levels of TMAO, is generally considered beneficial for health, indicating that the source and context of TMAO production might influence its effects. Contradictory findings, such as those from studies feeding rats a phospholipid–protein complex from Antarctic krill, further suggest that the relationship between TMAO, choline, and cardiovascular risk is complex and context-dependent. TMAO has also been proposed as a biomarker for metabolic syndrome, with elevated serum levels observed in patients with established cardiometabolic diseases [99,100].

### 4.29. MicroRNAs and Long Non-Coding RNAs

miRNAs are small, non-coding RNA molecules that play a crucial role in regulating gene expression. Their unique expression profiles and release patterns during various stages of cardiovascular diseases (CVDs) make them valuable biomarkers for diagnosis and prognosis. For heart failure, miRNAs such as miR-18, miR-37, miR-126, miR-210, miR-221, and miR-1254 are strongly indicative. Other significant miRNAs include miR-30d, miR-223-3p, miR-301a-3p, and miR-199a-3p, reflecting the diverse molecular mechanisms underlying heart failure progression. For acute myocardial infarction, miR-29b is a key marker, while miR-21, miR-208, miR-423-5p, and miR-499 are associated with both acute myocardial infarction and heart failure, providing insights into overlapping pathological pathways. MiRNAs like miR-23a, miR-26a, miR-150, and miR-483-5p are highly specific markers for arrhythmia, aiding in the diagnosis and understanding of arrhythmogenic mechanisms. Additionally, miR-1, miR-133, and miR-328 are implicated in acute myocardial infarction, arrhythmia, and heart failure, underscoring their central role in cardiovascular pathology [101,102,103]. The profiling of miRNAs offers valuable insights into the molecular pathways driving CVDs. These findings reveal potential therapeutic targets and provide tools for risk stratification and early diagnosis. The use of miRNAs as biomarkers holds significant promise in advancing personalized medicine for cardiovascular health. Table 4 indicates cardiovascular biomarkers with clinical relevance in diagnosis and prognosis. The “biomarker” column lists the specific biomarker being assessed for CVDs or inflammation. The “source” column identifies the primary cells or tissues responsible for the production of each biomarker. The half-life refers to the time it takes for the concentration of the biomarker to reduce by half in the bloodstream. Time window for detection indicates the period during which the biomarker is elevated, ranging from hours to days, depending on the condition. Specificity and sensitivity describe the accuracy of the biomarker in identifying cardiovascular diseases and their respective conditions, with sensitivity referring to its ability to detect disease, and specificity to its ability to identify only cardiovascular conditions. Factors affecting interpretation column outline various factors or conditions that may influence the levels of the biomarkers, such as other diseases, medications, or physiological states, that could compromise their clinical interpretations in practice.

## 5. Conclusions

This review highlights the pivotal role of cardiovascular biomarkers in understanding the pathophysiology, diagnosis, and prognosis of CVDs. Biomarkers such as cTns, CRP, BNP, and NT-proBNP are well-established tools in clinical practice, providing robust diagnostic and prognostic value for MI and HF. Emerging biomarkers like miRNAs, lncRNAs, and TMAO represent a breakthrough in molecular diagnostics, offering exceptional sensitivity and specificity for early disease detection, risk stratification, and therapeutic targeting. Enzyme markers, including AST, CK-MB, LDH, and HBDH, remain critical for detecting myocardial injury, especially during acute events. Protein markers, such as H-FABP and MPO, provide insights into inflammatory processes and oxidative stress, serving as indicators of both acute and chronic CVDs. Novel protein biomarkers like IMA and GPBB offer early signals of ischemic damage, augmenting the diagnostic repertoire. Peptide markers, including BNP and NT-proBNP, are indispensable in HF diagnosis and management, reflecting ventricular stress and myocardial remodeling. Innovative peptides such as MR-proANP and MR-proADM extend the spectrum of peptide biomarkers, providing additional insights into disease severity and progression. Lipid markers, including Lp-PLA2 and oxylipins, elucidate lipid metabolism’s contribution to atherosclerosis, while inflammatory biomarkers like TNFα, IL-6, GDF-15, and PTX3 highlight the role of chronic inflammation in CVD progression. Hormonal markers such as copeptin and ET-1 emphasize neurohormonal and hemodynamic stress pathways, while emerging candidates like ST2, Gal-3, PAPP-A, and TMAO provide valuable insights into fibrosis, myocardial remodeling, and metabolic dysregulation. Despite significant advancements, there remains a need for integrating novel biomarkers with existing clinical frameworks to enhance diagnostic accuracy and predictive capabilities. Emerging technologies like multi-omics approaches, AI-driven analytics, and point-of-care testing platforms hold immense promise for discovering new biomarkers and improving their clinical utility. In the future, the validation of miRNAs, lncRNAs, and metabolomic markers as routine diagnostic tools could significantly advance personalized cardiovascular care. Further research should focus on large-scale, multi-ethnic studies to validate biomarkers across diverse populations, ensuring equitable healthcare solutions. Additionally, exploring the role of biomarkers in subclinical CVD, identifying markers for early intervention, and investigating their potential in monitoring novel therapeutics could transform cardiovascular medicine. By leveraging innovative technologies and integrating biomarkers into personalized medicine, future advancements are poised to redefine the prevention, diagnosis, and management of CVDs, ultimately reducing the global disease burden.

## Figures and Tables

**Table 1:** Lipoprotein-associated phospholipase A2 (Lp-PLA2) is a biomarker of vascular inflammation and atherogenesis. It is synthesized by inflammatory cells in atherosclerotic plaques and binds to lipoproteins (HDL, LDL, and VLDL), promoting plaque progression and instability. Elevated Lp-PLA2 levels are linked to increased cardiovascular risk, providing diagnostic, prognostic, and therapeutic utility in CVDs.

Feature	Details
Biochemical role	Member of the phospholipase A2 superfamily; hydrolyzes oxidized phospholipids within lipoproteins, leading to the generation of proinflammatory mediators.
Primary source	Synthesized predominantly by inflammatory cells within atherosclerotic plaques.
Lipoprotein binding	Strong affinity for lipoprotein fractions, including HDL, LDL, and VLDL. This association highlights its role in lipid metabolism and atherogenesis.
Initial identification	Initially recognized as plasma platelet-activating factor acetylhydrolase (pPAF-AH) due to its ability to hydrolyze platelet-activating factor (PAF), a potent proinflammatory mediator.
Role in atherosclerosis	Contributes to vascular inflammation by hydrolyzing oxidized phospholipids in LDL, generating lysophosphatidylcholine and oxidized fatty acids, which promote plaque progression and instability.
Clinical significance	Elevated Lp-PLA2 levels are strongly associated with increased risk of cardiovascular diseases (CVDs), such as coronary heart disease (CHD), atherosclerosis, and ischemic stroke.
Diagnostic utility	Provides high specificity for vascular inflammation with minimal biological variability, making it a robust biomarker for assessing cardiovascular risk.
Prognostic implications	Elevated levels correlate with higher risks of cardiovascular events, plaque rupture, and future adverse outcomes.
Therapeutic potential	A potential therapeutic target due to its role in vascular inflammation and atherogenesis. Inhibitors of Lp-PLA2 are being investigated as possible interventions for reducing cardiovascular risk.
Inflammatory role	Mediates vascular inflammation through its regulatory effects on lipid metabolism and its proinflammatory activity within atherosclerotic plaques.
Utility in CVDs	Helps in diagnosing, prognosticating, and managing vascular diseases, particularly in patients predisposed to atherosclerosis, CHD, and ischemic stroke.
Advantages	High specificity for vascular inflammation; reflects the inflammatory state of atherosclerotic plaques; minimal biological variability.
Limitations	Elevated levels may reflect an inflammatory environment without necessarily pinpointing specific events, requiring integration with other clinical parameters for comprehensive assessment.

**Table 2:** Comparison of BNP and NT-proBNP are vital cardiac biomarkers used to diagnose, assess the prognosis of, and manage various cardiovascular diseases (CVDs). They are released in response to myocardial stretch and stress, making them sensitive indicators of cardiac dysfunction.

Feature	BNP	NT-proBNP
Source	Secreted by cardiac ventricles in response to stretch	Cleaved from proBNP during BNP synthesis
Molecular structure	Active hormone	Inactive fragment
Half-life	~20 min	~60–120 min
Stability	Less stable in blood samples	More stable in blood samples
Diagnostic role	Heart failure (HF) diagnosis, prognosis, and treatment monitoring	Heart failure diagnosis, prognosis, and treatment monitoring
Age impact	Less affected by age	Levels increase significantly with age
Renal impact	Moderately influenced by renal dysfunction	More significantly influenced by renal dysfunction
Reference ranges	<100 pg/mL typically indicates no HF	Age-specific cutoffs; <300 pg/mL often indicates no HF
Clinical use	Acute and chronic HF management	Acute and chronic HF management
Advantages	Directly reflects cardiac activity	Higher stability; useful for longer sample transport
Disadvantages	Short half-life, less stable	Age- and renal-dependent levels

**Table 3:** Comparison of MR-proANP and MR-proADM as cardiac biomarkers.

Feature	MR-proANP	MR-proADM
Biochemical source	Derived from the stable N-terminal portion of pro-atrial natriuretic peptide (proANP).	A stable fragment of adrenomedullin, a peptide involved in vasodilation, natriuresis, and diuresis.
Primary role	Reflects atrial stretch and fluid overload, primarily linked to heart failure (HF).	Reflects vascular stress, endothelial dysfunction, and cardiovascular stress.
Stability	Highly stable in circulation due to its longer half-life compared to atrial natriuretic peptide (ANP).	Highly stable, offering reliable measurement and prognostic insights.
Diagnostic utility	Effective for diagnosing heart failure (HF), although slightly less sensitive than BNP and NT-proBNP.	Useful in acute and chronic HF, acute coronary syndrome (ACS), and acute myocardial infarction (AMI). Adds diagnostic value beyond natriuretic peptides.
Prognostic utility	Excels in long-term prognostic value, particularly in predicting mortality over five years in chronic HF.	Superior prognostic value in predicting mortality and cardiovascular events in HF, ACS, and AMI. Outperforms natriuretic peptides in risk stratification for mild-to-moderate HF.
Heart failure (HF)	Strongly associated with disease severity and mortality in chronic HF; valuable for monitoring disease progression and treatment efficacy.	Levels correlate with HF severity and NYHA class; provides predictive value for long-term outcomes and mortality.
Acute coronary syndrome (ACS) and AMI	Limited data on utility in ACS and AMI.	Independently associated with fatal and nonfatal cardiovascular events in ACS and AMI. Enhances risk stratification beyond traditional models and natriuretic peptides.
Screening potential	Effective for screening atrial fibrillation (AF), particularly in community populations, and identifying individuals at risk of developing AF.	Primarily used for prognostic and risk stratification purposes; limited use in AF screening.
Influencing factors	Levels influenced by age, BMI, race, and sex; despite variability, it remains reliable due to its stability.	Levels independent of renal function, age, and systolic blood pressure; remains highly predictive despite other clinical variations.
Advantages	Long half-life, stable, and highly prognostic for HF and atrial fibrillation risk.	Adds significant prognostic value to traditional risk models, particularly in ACS and AMI; strong correlation with cardiovascular stress and mortality.
Limitations	Diagnostic sensitivity slightly lower than BNP and NT-proBNP; levels vary with demographic and physiological factors.	Limited availability in routine clinical practice; specific role in AF screening not established.
Clinical utility	Valuable in chronic HF management, long-term mortality prediction, and AF screening.	Crucial for prognosticating HF, ACS, and AMI outcomes, particularly in mild-to-moderate HF; enhances risk models for predicting mortality and cardiovascular events.

**Table 4:** Overview of cardiovascular biomarkers and their sources, half-life, and time windows for detection. It also includes details on their specificity, sensitivity, and factors affecting their clinical interpretation.

Biomarker	Source	Half-Life	Detection Time Window	Specificity and Sensitivity	Factors Affecting Interpretation
Aspartate aminotransferase (AST)	Cardiac muscle, liver, and skeletal muscle	17 ± 5 h	Rises in 6–12 h, peaks at 24–36 h, and normalizes in 3–7 days	Moderate sensitivity; low specificity for MI	Elevated in liver disease, muscle injury, and hemolysis
Creatine kinase MB (CK-MB)	Predominantly cardiac muscle and minor in skeletal muscle	~12–24 h	Rises in 3–6 h, peaks at 18–24 h, and normalizes in 48–72 h	High specificity for MI; lower sensitivity than troponins	Skeletal muscle injury, renal failure, and chronic muscle diseases
Lactate dehydrogenase (LDH)	Cardiac muscle and red blood cells (LDH1 > LDH2 ratio indicative of MI)	~9 h	Rises in 12–24 h, peaks in 2–3 days, and normalizes in 10–14 days	Low specificity; moderate sensitivity	Liver disease, hemolysis, malignancies, and strenuous exercise
Carbonic anhydrase III (CA-III)	Cardiac muscle and skeletal muscle	Not well established	Altered levels in oxidative stress and ischemia	Emerging biomarker; specificity/sensitivity under study	Ischemia, oxidative stress, and metabolic disorders
Heart-type fatty acid-binding protein (H-FABP)	Cardiac muscle	~1–2 h	Detectable within 90 min, peaks at 6–8 h, and normalizes in 24–36 h	High sensitivity for early MI detection; moderate specificity	Skeletal muscle injury and renal dysfunction
C-reactive protein (CRP)	Produced in response to cardiac inflammation, vascular endothelium	19 h	Increases within 6–12 h, peaks at 24–48 h, and remains elevated for days	High sensitivity for inflammation; low specificity for cardiac events	Chronic inflammation, infections, and autoimmune diseases
Myeloperoxidase (MPO)	Secreted by neutrophils and macrophages in atherosclerotic plaques	~12–24 h	Detectable early in ACS and remains elevated for several days	High sensitivity for oxidative stress; moderate specificity for MI	Infections, systemic inflammation, and autoimmune diseases
Cardiac troponins (cTnI, cTnT)	Cardiac myocytes (specific to heart muscle)	cTnI: 2 h–1 day, cTnT: 2–14 days	Rises in 2–4 h, peaks at 12–24 h, and persists for 7–14 days (cTnT)	Gold standard for MI; high specificity and sensitivity	Chronic renal disease, heart failure, sepsis, and strenuous exercise
Hydroxybutyrate dehydrogenase (HBDH)	Cardiac muscle and red blood cells	~10 h	Rises in 6–12 h, peaks at 48–72 h, and normalizes in 7–10 days	Moderate specificity; lower sensitivity than troponins	Liver disease, hemolysis, and muscle injury
Matrix metalloproteinases (MMPs) and TIMPs	Cardiac extracellular matrix and vascular smooth muscle cells	Hours to days	Chronically elevated in heart failure and atherosclerosis	High specificity for ECM remodeling; moderate sensitivity	Inflammation, cancer, and chronic heart diseases
Fibrinogen	Liver	~3–5 days	Elevated in the presence of inflammation or cardiovascular risk	Moderate specificity; increased in inflammatory states	Liver disease (synthesis affected), inflammation, and malignancies
Myoglobin	Skeletal and cardiac muscle	2–4 h	Detectable within 1–3 h, peaks at 6–9 h, and returns to baseline in 24 h	High sensitivity; low specificity	Skeletal muscle injury, renal failure, and exercise
Ischemia-modified albumin (IMA)	Serum albumin (modified by ischemia)	~6 min	Detectable within minutes and peaks within 3–6 h	Moderate sensitivity and specificity for ischemia	Liver disease, infections, and oxidative stress conditions
Glycogen phosphorylase isoenzyme BB (GPBB)	Cardiac and brain tissue	~1–3 h	Detectable within 1–4 h, peaks at 6–12 h, and normalizes in 24 h	High sensitivity; moderate specificity	Pregnancy, brain injury, and liver dysfunction
Oxylipins	Endothelial cells and inflammatory cells	Variable (minutes to hours)	Depends on lipid subtype	High specificity for endothelial function	Dietary intake and genetic polymorphisms
Lipoprotein-associated phospholipase A2 (Lp-PLA2)	Circulating with LDL and HDL	~6 days	Chronic marker	High specificity for vascular inflammation	Affected by lipid-lowering therapies and metabolic syndrome
B-type natriuretic peptide (BNP) and NT-proBNP	Cardiac myocytes	BNP: ~20 min; NT-proBNP: ~90 min	Detectable within hours and peaks at 24–48 h	High specificity and sensitivity for HF	Affected by age, renal function, and obesity
Mid-regional pro-atrial natriuretic peptide (MR-proANP)	Cardiac atria	~60 min	Detectable within hours, peaks within 24 h	High specificity, moderate sensitivity	Affected by renal dysfunction and age
Mid-regional pro-adrenomedullin (MR-proADM)	Endothelium, adrenal glands	~22 min	Detectable early in sepsis, HF	High sensitivity, moderate specificity	Affected by inflammation and sepsis
Endothelin-1 (ET-1)	Endothelium	~4–7 min	Detectable early in HF and hypertension	High specificity for endothelial dysfunction	Affected by renal disease and inflammatory conditions
Tumor necrosis factor-alpha (TNFα)	Immune cells	~30 min	Chronic marker	High sensitivity, low specificity	Elevated in systemic inflammation, and cancer, infections
Myoglobin	Skeletal and cardiac muscle	2–4 h	Detectable within 1–3 h, peaks at 6–9 h, and returns to baseline in 24 h	High sensitivity, low specificity	Skeletal muscle injury, renal failure, exercise
Ischemia-modified albumin (IMA)	Serum albumin (modified by ischemia)	~6 min	Detectable within minutes and peaks within 3–6 h	Moderate sensitivity and specificity for ischemia	Liver disease, infections, and oxidative stress conditions
Interleukin-6 (IL-6)	Immune cells and endothelial cells	~15–20 h	Peaks within 1–2 h and remains elevated for days	High sensitivity for inflammation, but low specificity for CVD alone	Chronic inflammatory diseases, infections, autoimmune disorders, and obesity
Growth differentiation factor-15 (GDF-15)	Cardiomyocytes and macrophages	~4–6 h	Elevated in hours to days during acute and chronic cardiovascular conditions	High sensitivity for heart failure; moderate specificity for CVD	Kidney disease, aging, cancer, and metabolic disorders
Suppression of tumorigenicity-2 (ST2)	Cardiac fibroblasts and immune cells	~1.5 h	Elevated in hours to days in acute and chronic heart failure; linked to adverse outcomes	High prognostic value in heart failure; moderate specificity for CAD	Infection, inflammatory diseases, and renal dysfunction
Pentraxin 3 (PTX3)	Endothelial cells, neutrophils, and macrophages	~12–48 h	Peaks within hours and stays elevated for up to several days during acute myocardial infarction	High sensitivity for acute inflammation; moderate specificity for CAD	Sepsis, autoimmune diseases, and infections
Pregnancy-associated plasma protein-A (PAPP-A)	Vascular smooth muscle cells and macrophages	~4 h	Elevated within hours to days in acute coronary syndromes and atherosclerosis progression	High specificity for unstable plaques; useful in CVD risk stratification	Renal dysfunction, pregnancy, and chronic inflammation
Copeptin	Secreted in equimolar amounts with AVP, from the posterior pituitary gland	1–2 h	Within hours of acute myocardial infarction (AMI) onset; peaks on 1st day	High sensitivity for early AMI detection; good negative predictive value	Kidney function, age, and comorbidities like diabetes may affect copeptin levels.
Galectin-3 (Gal-3)	Secreted by activated macrophages, linked to fibrosis, inflammation, and remodeling	~1–2 days	Elevated in both acute and chronic heart failure (HF); often detectable early	Moderate specificity for HF; high sensitivity for fibrosis and inflammation	Renal dysfunction can elevate levels; interpretation may vary with concurrent inflammation.
Trimethylamine N-oxide (TMAO)	Produced by gut microbiota from l-carnitine and choline metabolism	Several hours to days	Can be detected after consumption of dietary choline or l-carnitine, early in atherosclerosis	Moderate sensitivity for cardiovascular risk; linked to poor prognosis in CVD	Dietary intake, gut microbiota composition, and kidney function can influence TMAO levels.
MicroRNAs (miRNAs)	Released from cardiomyocytes, endothelial cells, and other cells during cardiovascular stress	Varies (hours to days)	Can be detected within hours of AMI or heart failure onset	High specificity for distinct CVDs; good sensitivity for early detection	Stable in plasma, but affected by collection and processing methods. Interference from other conditions can complicate interpretation.

## Data Availability

The data presented in this study are available upon request from the corresponding author. The data are not publicly available due to privacy restrictions.
